# Mid-Term Results of an Italian Multicentric Experience with the Roadsaver^TM^ Dual-Layer Carotid Stent System

**DOI:** 10.3390/healthcare12010120

**Published:** 2024-01-04

**Authors:** Olga Silvestri, Giulio Accarino, Davide Turchino, Francesco Squizzato, Michele Piazza, Martina Bastianon, Sara Di Gregorio, Giovanni Pratesi, Michele Antonello, Davide Costa, Raffaele Serra, Umberto Marcello Bracale

**Affiliations:** 1 Department of Public Health, Vascular Surgery Unit, University Federico II of Naples, 80131 Naples, Italy; olga.silvestri@unina.it (O.S.); g.accarino@live.it (G.A.); umbertomarcello.bracale@unina.it (U.M.B.); 2 Department of Medicine, Surgery and Dentistry, University of Salerno, 84084 Fisciano, Italy; 3 Department of Cardiac, Thoracic and Vascular Sciences and Public Health, School of Medicine, Padua University Hospital, 35100 Padua, Italy; francesco_squizzato@icloud.com (F.S.); michele.piazza@unipd.it (M.P.); michele.antonello.1@unipd.it (M.A.); 4 Vascular and Endovascular Surgery, IRCCS Ospedale Policlinico San Martino, University of Genova, 16100 Genova, Italy; 5120886@studenti.unige.it (M.B.); sara.digregorio874@gmail.com (S.D.G.); giovanni.pratesi@unige.it (G.P.); 5 Interuniversity Center of Phlebolymphology (CIFL), International Research and Educational Program in Clinical and Experimental Biotechnology, University Magna Graecia of Catanzaro, 88100 Catanzaro, Italy; rserra@unicz.it

**Keywords:** carotid artery stenosis, carotid artery stenting, dual-layer stent, micromesh, stroke

## Abstract

Background: Carotid artery stenting (CAS) using first-generation single-layer stents is widely accepted as a good alternative to standard carotid endarterectomy (CEA) but it is associated with worse outcomes in terms of both plaque prolapse and cerebral embolization. Aim: To evaluate the perioperative and midterm outcomes of CAS using the new-generation Roadsaver^TM^ dual-layer micromesh-covered carotid stent. Methods: Herein, we present the results of an observational, retrospective, multicentric study on non-consecutive patients who underwent the CAS procedure between January 2017 and December 2022 at three Italian, high-volume vascular surgery centers. The inclusion criteria were the patients’ eligibility for the CAS procedure in accordance with the current Italian guidelines, and the implantation of a Roadsaver stent. Both symptomatic and asymptomatic patients were included in the study. The patients requiring reintervention for carotid restenosis following CEA were also included. Perioperative data regarding procedural success was defined as the successful implantation of the device in the desired position, less than 30% residual stenosis, and the absence of intraoperative neurological complications. The primary outcome was any adverse cerebrovascular event such as stroke or transient ischemic attack (TIA) during the procedure and/or after discharge. The secondary outcomes were the need for further intervention, and all-cause death following procedure. Results: Three-hundred-fifty-three (353) patients were included in our study; the mean age was 74.3 years. A total of 5.9% of the patients were symptomatic on their operated side, while 7.3% had contralateral carotid occlusion. A cerebral embolic protection device (CPD) was employed in all patients. A total of 13.3% of the patients were operated on for restenosis after CEA Technical success was achieved in 96.9% of the cases with an intraoperative report of six TIAs (1.7%) and six ipsilateral strokes (1.7%). The mean hospital stay was 1.8 days. The thirty-day follow up showed one TIA and one more stroke. At the mean 35-month follow-up time, the primary outcome was present in six patients (1.7%), where four TIAs (1.1%) and two strokes (0.5%) were reported. Restenosis occurred in five patients (1.4%). Death for any cause was reported in 11 patients (3.1%). Conclusions: As most recent, high-quality studies show, the CAS procedure with second-generation devices such as the Roadsaver stent is safe and effective in preventing carotid-related cerebrovascular events in both symptomatic and asymptomatic patients. The intraoperative and postoperative cerebrovascular complication rate in high volume centers is very low, ensuring confidence in its employment for the CAS procedure along with a CPD as a valid alternative to CEA.

## 1. Introduction

Carotid artery stenting (CAS) was introduced as an alternative to carotid endarterectomy (CEA) to treat high-surgical-risk patients or those having specific contraindications to CEA [1]. However, CAS has been linked to higher perioperative cerebral risk compared with CEA [1] and, therefore, indications for CAS have been limited to restenosis following CEA, previous radiation therapy or neck surgery, contralateral laryngeal nerve palsy, and patients with prohibitive surgical risk. 

Two types of complications can be distinguished: an intraoperative one with extremely rapid onset and a late one that emerges during follow-up. One of the most fearsome risks is intraprocedural embolization; the specific risk of embolization is still debatable and depends on a number of variables, such as air embolization, thrombus dislodgement, or plaque fragmentation from the catheter, as well as manipulation of the wire or sheath in the common carotid or aortic arch. Embolization and ischemic events have also been reported outside of the vascular territory of the treated ICA. Additional periprocedural risks include the possibility of dissection of the treated segment and early stent thrombosis. The former can be avoided by performing very gentle maneuvers while the latter has been greatly reduced since the introduction of dual antiplatelet therapy. Finally, complications related to femoral access can occur but are much less serious [2]. Late complications can include stent thrombosis, frequent if medical therapy is discontinued prematurely, restenosis, and stent fracture. Restenosis after CAS is still higher than after CEA; however, it is tied to poor initial anatomical results and is therefore predictable [3]. It is, however, well-established that balloon angioplasty without stent implantation carries a high dissection risk and should be avoided [4]. 

Today, CAS has become a valid alternative in the treatment of carotid artery disease in many centers due to its minimally invasive nature and proven patient satisfaction. 

The CREST trial in patients with symptomatic and asymptomatic carotid artery stenosis confirmed similar outcomes for CAS and CEA at their primary endpoints (stroke, death, myocardial infarction) but showed a relative increase in minor strokes within 30 days—mainly post-procedural—following CAS [5]. The data reported in the previous study and in the latest guidelines refers mainly to first-generation stents and are derived from comparisons between closed-cell or open-cell stents [6,7]. Growing experience on dual-layer stents has led the European Society for Vascular Surgery (ESVS) to indicate that for patients undergoing elective carotid artery stenting, dual layer mesh-covered stents (DLS) may be considered [8]. 

The advent of Cerebral Protection Devices (CPDs) has greatly reduced stroke risk related to stent positioning and inflation, as proven by the SAPPHIRE trial [9].

The use of a CPD is currently recommended in all vascular surgical guidelines [10], as the reduction in adverse cerebral events following the use of these devices suggests a correlation between the design of the stents used and the containment of plaque protrusion through the stent. Procedures in which CPDs were not used showed a 4-fold increase in the likelihood of intraoperative stroke [11]. 

The first carotid balloon angioplasty procedure was published in 1980 [12]. Since its inception, the technique has been always considered high-risk for its potential of dissection, while the first carotid artery stenting was published in 1989 to treat an intimal flap consequent to a carotid angioplasty [12]. The first available devices were balloon-expandable stents derived from the Palmaz stent and, as they were subject to crushing/collapsing in up to 15% of cases, they were not commonly used in routine practice [13]. The use of the auto-expandable Wallstent series, originally meant for the iliac and peripheral district, marked the end of the balloon-expandable stents and resolved the compression and twisting problem. The first device designed to be used in the carotid artery was the Carotid Wallstent (Boston Scientific Corporation, Marlborough, MA, USA) made from a cobalt alloy and is still used today as a closed cell stent [14]. Most of the currently available carotid stents are made of nitinol, a nickel–titanium alloy currently widely utilized in the endovascular market for its thermally dependent shape-memory proprieties. These proprieties allow for the selection of the radial force of the device according to its nominal diameter, paving the way for the production of stents with various shapes, including tapered stents to account for the difference in diameter between the ICA and the common carotid artery ensuring a homogenous radial force. Both closed and open cell stents carry advantages and pitfalls, as their architecture has been demonstrated to be effective in different anatomies [15]. Nevertheless, plaque scaffolding, accuracy during delivery and long-term safety have been a challenge for years. 

Sophisticated stent design has begun to represent turning points in reducing the complications arising from CAS due to the fact that the choice of stent depends on the configuration of the stent itself and on the anatomical features of the artery as well as the particular characteristics of the lesion to be treated.

Commercially available self-expanding carotid stents are composed of either nitinol (a nickel–titanium alloy) or stainless steel (a cobalt alloy). Once deployed in the body, nitinol stents rely on their thermal memory to achieve their predefined shape. In addition to their material composition, stents can also be classified according to their structure. They can be of a single layer, and can have closed or open cells or a hybrid design [16]. 

This feature allows for the containment of residual plaque, the limiting of plaque prolapse, and guarantees that no debris is dislodged through the stent meshes [17]. Another important consideration in device selection is the operator’s handling and experience with the stent being used. It has been well-documented in multiple studies that high-volume providers are a predictor of better CEA outcomes [18,19]. 

Different studies have demonstrated that large-volume centers performing CAS was an important predictor of outcome. Providers who have a low annual volume of CAS procedures have higher rates of MAEs [20]. The aim of our study was to assess the safety and effectiveness of CAS in a real world setting on patients treated in three high-volume Italian centers with the Roadsaver stent and CPD.

## 2. Materials and Methods

We retrospectively reviewed a consistently maintained registry of 353 patients with atherosclerotic carotid disease treated with the Roadsaver stent (Terumo Europe NV, Leuven, Belgium) in three high-volume Italian centers between January 2017 and December 2022. 

The Roadsaver Carotid Stent is a nitinol-based self-expanding stent intended primarily for use in carotid artery stenting procedures. It has a mesh configuration that distinguishes it from traditional open or closed cell stents. The stent is constructed around a single, self-expanding nitinol wire that has a closed-cell design at the distal end and an open-cell design at the proximal end. This combination of open and closed cells creates a stable and adaptable framework for carotid artery lesion stenting and scaffolding. The RoadSaver Stent’s closed-cell portion provides stability and support which is critical in regions of the carotid artery that are prone to plaque rupture. Its low 5Fr profile gives accurate insertion in narrow lesions, reducing the danger of embolization during deployment. The open-cell architecture allows for conformability and adaptation to the various carotid artery anatomies. This adaptability is combined with the possibility of retrieving the device up to 50% of its deployment for optimal positioning over the carotid plaque. 

All principles of the Declaration of Helsinki were adhered to in our study as well as conformance with Italian privacy laws (Art. 20–21, DL 196/2003) as published in the *Official Journal*, volume 190, 14 August 2004, which explicitly waives the need for ethical approval of the use of anonymous data. The study was approved by the Institutional Review Board of Interuniversity Center of Phlebolymphology (CIFL), and the International Research and Educational Program in Clinical and Experimental Biotechnology (Approval number: ER.ALL.2018.68A.).

All patients gave their written consent for the anonymous collection of clinical data. At time of treatment, patients’ demographics, comorbidities, and clinical history were systematically collected. Inclusion criteria were the presence of a bulbar or internal carotid artery (ICA) stenosis between 50% and 99% in the presence of previous consistent symptoms, transient ischemic attack (TIA) or amaurosis fugax, verified as consistent with a carotid stenosis, minor or major ipsilateral stroke or asymptomatic with stenosis between 70% and 99%. Stenting was preferred to traditional open treatment in patients with contralateral carotid artery occlusion or patients deemed at very high surgical risk in the presence of a contralateral laryngeal nerve palsy, previous ipsilateral neck surgery, previous radiant therapy or treatment for carotid restenosis. All grades of stenosis were calculated by a duplex ultrasound (DUS) examination according to the NASCET (North American Symptomatic Carotid Endarterectomy Trial) criteria [21]. To assess the structure of the aortic arch, the angle of the supra-aortic arteries and any accompanying intracranial lesions, computed tomography angiography or magnetic resonance imaging were required for all patients. Patients with type II and III aortic arch were excluded. All patients were prescribed dual antiplatelet therapy before endovascular treatment which was maintained subsequently according to guidelines. 

All procedures were performed using a percutaneous femoral access under local anesthesia. Intravenous heparin was administered at a dose of 70 U/kg and a CPD was used for all patients. After discharge, patients were prescribed dual antiplatelet therapy and were followed up at 1, 3, and 6 months after treatment and yearly thereafter, with DUS examination and clinical evaluation. Patients were instructed to notify the referring institution in case of new-onset neurological symptoms. Patients were also contacted by the referring institution for information on their post-procedural course and current follow-up. Primary endpoint of the study was a cerebrovascular event as a TIA, a minor or major stroke as defined by reporting standards consistent with the operated carotid artery periprocedurally and at the longest available follow-up. Secondary outcomes included death from any cause and carotid restenosis. Procedural success was defined as a less than 30% residual stenosis, correct positioning of the stent, and the absence of intraoperative neurological complications. Statistical analysis was carried out with version 1.4.1106 © 2009–2021 RStudio, PBC. The Kaplan–Meier method was used to evaluate time-to-event data.

## 3. Results

Between January 2017 and December 2022, a total of 353 patients were treated with the Roadsaver stent (Terumo Europe NV, Leuven, Belgium) in three high-volume Italian centers located in Padua, Genoa, and Naples. The patients’ demographic and comorbidities are reported in Table 1. The mean age at the time of the treatment was 74.3 ± 8.3. 

The most common comorbidities were hypertension, present in 316 out of 353 patients (89.5%) and dyslipidemia in 292 (82.7%) patients. Twenty-one (5.9%) patients were symptomatic, while 27 (7.6%) presented a contralateral carotid occlusion. The procedural features are reported in Table 1. Cerebral protection was used for all cases. Predilatation before stent insertion was not necessary for any of the patients. 

The most frequently used cerebral protection device was the Spider FX (Medtronic Inc., Minneapolis, MN, USA), employed in 45% of the cases. 

The other CPDs used were the Robin (W. L. Gore & Associates; Newark, DE, USA) in 29.1% of the patients, Filter Wire EZ (Boston Scientific, Marlborough, MA, USA) in 16.7% of the patients; Emboshield NAV (Abbott Vascular, Redwood City, CA, USA) in 7%, and MoMa (Medtronic/Invatec, Santa Rosa, CA, USA) used in 1.9% of the patients.

### 3.1. In-Hospital Outcomes

Cerebrovascular intraoperative events were reported in 10 (2.83%) patients (six TIAs, four strokes) Table 2. Among the patients who reported neurological events, three of these were a major stroke and one was a minor stroke. Procedural success was achieved in 98.5% of the cases. One case of transient contrast-induced was reported and the patient did not require any further treatment. Comparing the intraoperative stroke rates divided by the type of CPD used, we noticed a statistically significant difference between the proximal and distal devices with two intraoperative cerebrovascular events in the seven patients treated with the proximal CPD (*p* = 0.014). No periprocedural myocardial infarctions were reported. Five patients had a MACE during follow-up after at least 3 months. The length of stay was 1.8 ± 0.7 days. No in-hospital deaths were reported.

### 3.2. Thirty-Day and Medium-Term Outcomes

While one TIA and one stroke at 30 days’ post-procedure occurred in two patients (0.56%), no early (90 days) deaths were reported. The mean follow-up time was 35 months, with 92 patients exceeding 48 months. At the last available follow up, four more TIAs and two more strokes (1.1 and 0.5%, respectively) were reported with only five (1.4%) restenoses, one of which resulted in an asymptomatic occlusion. All of these were treated. 

**Figure 1:**
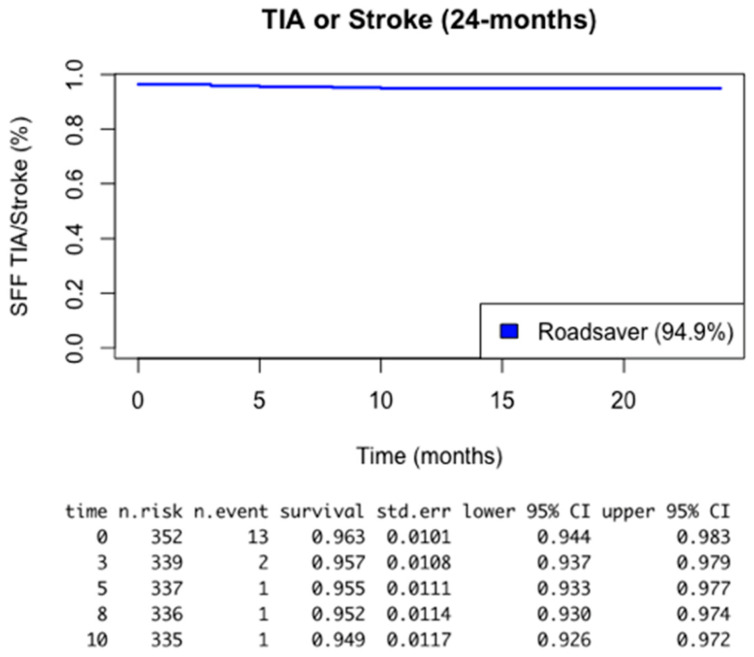
Kaplan–Meier analysis of 24 months survival free from TIA or stroke.

**Figure 2:**
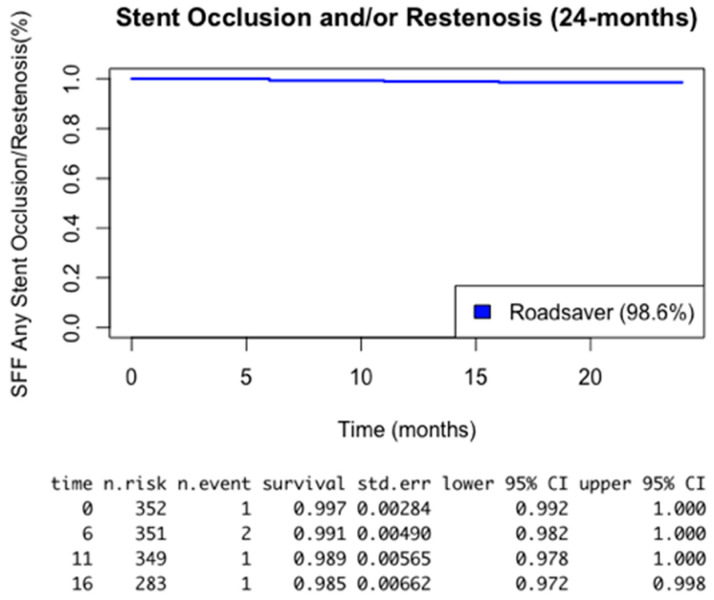
Kaplan–Meier analysis of 24 months survival free from stent occlusion and/or restenosis.

To summarize, a Kaplan–Meier analysis at 24 months reported a 94.9% survival free from TIA/stroke and a 98.6% survival free from restenosis/occlusion, respectively (Figure 1 and Figure 2). No deaths related to the procedure or to the operated ICA were reported. All-cause deaths occurred in 11 (3.1%) cases after a median time of 20 months (12.5–23.5).

The primary outcome was present in 18 (5.1%) of the patients and the secondary outcome was present in 16 (4.53%) of the patients. 

## 4. Discussion

To the best of our knowledge, herein, we report on one of the largest studies undertaken using the dual-layer micromesh-covered carotid stent system Roadsaver. The short- and long-term outcomes of carotid stenting procedures are closely related to the experience of the individual operator [22,23] which some studies report requires a long learning curve [24]. Even though carotid stenting showed great promise, its intraoperative neurological complication rate quickly proved to be higher than that of traditional surgical technique [9]. 

First-generation devices in the CREST trial were not able to prove the superiority of CAS over CEA in the composite outcome and, furthermore, did show more cerebrovascular adverse events in the CAS treatment study arm [25]. Consequently, CAS became elective for patients who could not undergo traditional open surgery [8]. The introduction of newly developed devices to the market which tended to overcome the limitations of first-generation devices significantly changed neurological outcomes [26] while maintaining the reduction in postoperative cardiac events guaranteed by CAS over CEA, suggesting a forthcoming paradigm shift. 

Part of the credit for the improvement of the CAS results should be attributed to the widespread use of ever better CPDs. A recent network meta-analysis by Giannopulos et al. [27] failed to identify any statistically significant difference in the cerebrovascular outcomes between different filters but further highlights the importance of a CPD of any kind when performing CAS. In our multicentric experience, five CPDs have been used, with the Spider FX being the most employed in 45% of the cases, followed by Robin (29%) and FilterWire (16%). All the CPDs used, with the exception of Mo.Ma, are essentially dedicated guidewires with a nitinol filtering basket on the distal edge that should be opened in the distal ICA and aims to capture any debris moving from the carotid and the aortic arch during the procedure. The Mo.Ma, on the other hand, bases its operation on halting the ICA blood flow with a double balloon that must be inflated in the external and common carotid artery (CCA), respectively. A study published in February 2020 compared the Carotid Wallstent Versus Roadsaver Stent and Distal Versus Proximal Protection on Cerebral Microembolization and concluded that the Roadsaver stent and Mo.Ma significantly reduced the microembolic signals on a transcranial Doppler [28]. 

A recent and innovative technique for performing CAS using flow reversal is Transcarotid Artery Revascularization (TCAR). TCAR involves stent positioning via direct transcervical carotid access that may be performed with local anesthesia. TCAR eliminates the risks derived from arch manipulation and is particularly useful in patients with hostile aortic arch anatomy. During TCAR-based CAS, the dedicated introducer is placed in the CCA and by common femoral vein puncture, establishes flow reversal in the ICA and CCA, giving complete embolic protection [29]. Although very promising, no randomized controlled trial has yet compared TCAR to CEA or transfemoral CAS; the quality of the currently available data is modest and derived mostly from single-arm industry-sponsored studies and lacks a clear distinction between symptomatic and asymptomatic patients [30]. 

Current single-center experiences report a fairly low rate of neurological sequalae and optimal patient satisfaction for the noninvasive CAS procedure [31]. Some of the risks related to the endovascular procedure are inherent in the procedure itself in that they involve passing multiple devices through the aortic arch which could be exposed to damage. In fact, the manipulation of these arterial segments by guides and catheters is responsible for a high percentage of intraoperative strokes during carotid stenting [32]. 

In addition to the aforementioned risks, the difficulty in stent selection must be considered, being that a closed-cell stent is often unable to perfectly scaffold the irregular plaque wall, while an open-cell stent risks cutting the plaque and allowing the spread of microemboli [33]. For a long time, the choice of device to use was based on the physical characteristics of the individual plaque [34], whereby unstable plaques with greater soft components had a significant tendency to rupture through the stent mesh, while more calcific plaques often led to the stent not being in contact with the plaque for the intended length [35]. It is evident that vascular surgeons approaching carotid stenting are in need of a more versatile solution to safely treat a wider range of carotid plaques and feel confident in doing so. 

The mesh design of the Roadsaver Stent is a compromise between open and closed cells, providing superior conformability to the plaque surface. This versatility enables improved lesion coverage, lowering the risk of plaque disruption and embolization during deployment and after implant.

The optical coherence tomography (OCT) technique has enabled new evaluation possibilities for patients undergoing CAS. OCT can easily identify stent malapposition, plaque prolapse, and overall plaque scaffolding [36]. The mesh pore aperture size of the roadsaver stent is 375–500 μm. This limits intra- and post-operative plaque prolapse determined by the “cheese-grater effect” [37]. Different mesh-covered stents have shown different profiles of apposition and different risks of plaque prolapse [36]. 

The closed-cell part of the RoadSaver Carotid Stent offers a strong framework at the distal end, as plaque embolization during stent deployment is reduced and micro movements are limited during device retrieval. The structural integrity of the device construed from a single filament reduces the danger of device movement and assures the proper covering of the stenotic lesion, while its open-cell design allows it to adapt to a wide range of carotid artery anatomies. It is worth highlighting that the Roadsaver stent can also be repositioned until it is 50% deployed, further improving selective lesion treatment even by non-experienced surgeons. 

Because of its adaptability, the Roadsaver Stent may be used in a broader range of instances, possibly reducing the need for the choice of stent design and simplifying the selection process for surgeons. One of the possible drawbacks reported with this technology, however, is the fact that multiple metal layers exert a higher-than-average radial force on the vessel wall than other single layer devices, which could contribute to increased episodes of hypotension during and after the procedure [38] but may be controlled by a thorough monitoring of the patient’s blood pressure values. 

Many authors have confirmed that the perioperative risk with the device is less than 2% and that the stroke-free survival at 4 years following the procedure is circa 90%. Stabile et al. asserted that the use of carotid DLS correlated with a low rate of periprocedural adverse events which could possibly nearly eliminate post-procedural adverse events independent of clinical, anatomic, and procedural characteristics [25]. 

This is consistent with the presumed embolic prevention efficacy of the DLS during the stent healing phase and might eventually result in a clinically relevant benefit in relation to conventional carotid stents. Pini et al., in their review and meta-analysis, suggested that the overall 30-day stroke rate in asymptomatic patients was 1.4%, with a low level of heterogeneity in the cohort, and theorized that this low stroke rate may have been driven by the asymptomatic patients. By comparing the two stents with similar characteristics, they ascertained that the two types of DLS stents had a similar outcome (0.9% CGuard and 2.2% Roadsaver/Casper, *p* = 0.28). Pini also evaluated symptomatic patients with a 30-day stroke rate as low as 1.9% in 525 patients [1]. 

The current guidelines, supported by Imamura’s study [1], recommend, albeit supported by a low level of evidence, consider DLS for treating elective patients with carotid stenosis, further highlighting the importance of this valuable tool. In our experience, 21 (5.9%) of the patients subjected to CAS were symptomatic and only one had suffered periprocedural stroke.

To summarize, the main findings of our 5-year, 353 patient study are as follows:

- The reporting of an intraoperative complication rate of less than 1.7% using the Roadsaver stent associated with a CPD;
- Reporting, at an average follow-up of 35 months, a cumulative stroke rate of 2.2% and a transient ischemic attack rate of 2.8%; lower than many reported experiences in the literature, as indicated in Figure 1.

### 4.1. Future Research

Our conclusive findings give us the perspective pointing to possible directions for future research:

- To assess more accurately the extent to which brain protective devices may interfere with the number of intraoperative strokes
- To evaluate transcervical carotid access as a safer and simpler alternative to femoral artery access
- To assess more accurately the origin and evolution of the morphology for each type of plaque following implantation of different types of stents and whether this may have any influence on the degree of restenosis and distant postoperative events.

### 4.2. Study Limitations

The limitations of the study are the low number of cerebrovascular events which makes performing multivariate analyses aimed at finding possible relationships with cerebrovascular events virtually impossible, and an absence of a control group. The nonconsecutive nature of the patients enrolled, possible differences between the operators’ experience with CAS, which were not evaluated, an absence of intraoperative monitoring related to the stent placement, the exclusion by one of the centers of patients with arch type II and III that determined the retrospective analysis of only non-hostile aortic arch anatomies, which are known to experience worse outcomes and more complicated procedures with transfemoral stenting, and the lack of an exact division of cerebrovascular events in the assessment of the outcomes could also be considered as study limitations.

## 5. Conclusions

Based upon our experience and as supported by the short- and mid-term results of our study, we can assert that carotid stenting with the Roadsaver stent appears to be safe and effective in both symptomatic and asymptomatic patients, retaining an acceptable level of complications including patient discomfort and wound-related complications commonly related to open surgery. Notwithstanding our study limitations, the current results on this fairly new technology are encouraging and the accurate selection of patients to undergo CAS is fundamental in assuring good outcomes. Further studies such as ours will help to define exactly how recent technological advancements in stent design have improved the perioperative and long-term outcomes and will also allow for the better personalization of surgical treatment for each individual patient.

## Figures and Tables

**Table 1:** Subject clinical and procedural characteristics. SAP = systolic arterial blood pressure; DAP = diastolic arterial blood pressure; HD= hypoglycemic drugs; TGLs = triglycerides; LLDs = lipid-lowering-drugs; CAD = coronary artery disease; CKD = chronic kidney disease; GFR = glomerular filtration rate.

	Overall (*n* = 353)
Subject clinical characteristics	
Age (years)	74.3 ± 8.3
Males	259 (73.3%)
Smoke	206 (58.3%)
Hypertension (SAP > 140 mmHg and/or DAP > 90 mmHg)	316 (89.5%)
Diabetes (glycemia > 125 mg/dL and/or use of HD/insulin)	130 (36.8%)
Dyslipidemia (Tot. Chol. > 240 mg/dL and/or TGL > 150 mg/dL and/or use of LLD)	292 (82.7%)
CAD	148 (41.9%)
CKD (GFR < 60 mL/min/1.73 m^2^)	48 (13.6%)
End-Stage Kidney Failure (intra- or extracorporeal dialysis)	4 (1.4%)
Symptomatic stenosis	21 (5.9%)
Contralateral carotid occlusion	27 (7.6%)
Procedural characteristics	
Procedure for carotid restenosis	47 (13.3%)
Stent length (mm)	23.1 ± 4.1
Stent diameter (mm)	7.7 ± 0.6
Cerebral Protection System (CPD):	
- Spider Fx	159 (45%)
- Robin	103 (29.1%)
- Filter Wire EZ	59 (16.7%)
- Emboshield NAV6	25 (7%)
- Moma	7 (1.9%)

**Table 2:** Safety and effectiveness outcomes (24 months).

	Overall (*n* = 353)
Safety and Effectiveness Outcomes (24 months)	
TIA:	10 (2.8%)
- Intra operative	6 (1.7%)
- Post discharge	4 (1.1%)
Stroke:	8 (2.2%)
- Intra operative	6 (1.7%)
- Post discharge	2 (0.5%)
Restenosis	5 (1.4%)
All-cause death	11 (3.1%)
Hospital stay (days)	1.8 ± 0.7

## Data Availability

Data used and analyzed during the current study are available from the corresponding author upon reasonable request.
